# Open-Air Hospitals in War-Time

**Published:** 1914-09-19

**Authors:** Robert Saundby

**Affiliations:** Professor of Medicine, Birmingham University


### OPEN-AIR HOSPITALS IN WAR-TIME.

By ROBERT SAUNDBY, M.D., F.R.C.P., Professor of Medicine, Birmingham University.

BUILDING committees have been in the past anxious to erect a structure which as they say should give those who subscribe to its cost something to look at and be proud of. Such sentiments have to my knowledge greatly influenced the builders of hospitals, and have been put forward as the justification for a good deal of not strictly necessary ornament. During the last twenty years we have been learning the lesson slowly, but it is sinking in deeply, that the usefulness of hospitals is almost in inverse proportion to their architectural merits, and that our exaggerated notion of "comfort" is inconsistent with healthy surroundings. Draughts of air, low atmospheric temperature, dampness are not the disease-bearing agencies they were once supposed to be, while bad ventilation and equable, warm temperatures are depressing, debilitating, and retard recovery. Even dampness does no harm where there is a good current of air.

### THE DANGERS OF ARTIFICIAL VENTILATION.

With the elaborate artificial system of ventilation introduced into the Birmingham General Hospital it was possible to maintain the wards and corridors at practically the same temperature day and night, and this was fixed at 60° F. as the recognised sick-room figure. But it was soon found that its very equability was a disadvantage, that those who had to work in these temperatures felt weakened, and within a few months after the building was opened for use the temperature of the corridors and that of wards at night was reduced to 55° F., the former figure of 60° being retained for the wards by day. By degrees complaints were heard, not from those who had to work in these temperatures or on their own account, but by and on behalf of the patients, especially the surgical patients, and above all the children, complaints of delayed convalescence, of debility, and of retarded healing of wounds, results which would have been more seriously felt and less easily borne if it were not the case that the hospital has in the Jaffray branch a suburban dependency to which stationary cases can be transferred, thus relieving the beds of the parent institution. In spite of these advantages the complaints did not diminish and the need for fresh air was urgently felt. By many members of the staff the elaborate system of ventilation was regarded as worse than a failure, but as it provided the only means of heating the building it was endured, and other means of remedying the evils were sought.

### THE BETTER WAY.

Fortunately the architects had provided balconies, more for ornament than utility, at the free ends of the wards, and to these balconies, enlarged in some cases by structural alterations, the patients are removed, being accommodated in long wicker chairs; but even so only a proportion of the patients can benefit by this treatment. A proposal to build large verandahs to each of the wards of the Jaffray Branch, to which access should be given by windows opening down to the ground, was, although supported by the medical staff, rejected by the committee on the grounds of structural difficulties and expense, and this highly desirable alteration still remains to be made.

### AN OPEN-AIR WARD.

It was at the Queen's Hospital that the first attempt was made, although only on a small scale, to treat ordinary hospital patients in an open-air ward. The ward is on the roof of the hospital and is about half covered in, but quite open to the south. It certainly did not look "comfortable," and seemed open to the objection of getting all the smoke as well as the rain and snow. After it had been in use for some time I visited it with the late Professor Foxwell, who had suggested its creation, and what surprised me most was that patients and nurses seemed to like it, and my fears that it would be regarded as "uncomfortable" received no kind of encouragement from those to whom I spoke. Moreover, I found that they were not surgical cases only, but in fact were chiefly medical, and in particular I was shown a case of pneumonia which was doing very well. I climbed down from that visit to the roof converted, not to the advantages of open-air treatment only, for of that I had been long convinced, but of the practicability of carrying it out even in the middle of a smoky city like Birmingham.

### EXPOSED TO SUN, WIND, AND WEATHER.

During the meeting of the British Medical Association in Liverpool in 1911 I had the pleasure of visiting the Country Hospital for Children, where, in a large ward quite open to the estuary of the Dee, I found something like 150 children of ages from two to ten or thereabouts. Their cots were placed close together and in three rows parallel with the long axis of the ward and to the open side. Here there was no question of floor space or cubic feet of air. They were in the fresh air, exposed to all the sun that shone on that shore, and to all the wind and weather that might befall. These children from the slums of Liverpool were suffering from all sorts of diseases, medical and surgical, and were under the care of Dr. Macalister and Mr. Robert Jones. They were presumably ill, but they did not look it. They had rosy cheeks for the most part, and their appearance testified most strikingly to the vivifying effect of open air. They were as unlike the ordinary inmates of a children's hospital ward as it is possible to imagine. Why have we been slow to recognise that fresh air is the best tonic, the best antiseptic? It is cheaper, pleasanter, and undoubtedly more efficient than drugs. The successful treatment of tuberculosis in the open air led the way to the wider adoption of this method for other diseases. It was his observation of the results achieved by Dr. Huggard at Davos, whose advocacy of fresh air and courage and success in inducing his patients to discard their prejudices in favour of "warmth and comfort" were so remarkable, that inspired Dr. Foxwell with the wish to give the same plan a trial in the treatment of ordinary diseases at the Queen's Hospital. Yet the open-air treatment of consumption has been carried out for only some twenty-five years, although its value had been demonstrated by Mr. George Bodington, of Sutton Coldfield, and explained by him in his little book on "The Treatment of Pulmonary Consumption," published in 1840. Half a century had to pass away before the plan was revived, and this was not the direct result of Bodington's teaching; nor, we must regret to feel, was it due to the efforts or clear-sightedness of his own countrymen. The Warwickshire surgeon might know the truth, but his little lamp soon flickered out, and his contribution to the knowledge of his profession was forgotten, although when its value was proved by the work of Walther of Nordrach and others it received the belated honour of being included in the 173rd volume of the New Sydenham Society's reprinted monographs (1901).

### The American Civil War Results.

The experience of the American Civil War was all in favour of improvised hospitals or tents, but it preceded the enormous advance in surgical treatment due to Listerism, so that surgeons look back on those days with a shudder at results which were so poor, even where there was abundance of fresh air and sunlight, owing to the existing ignorance of the causes of wound infection. Yet those who took part in the medical and surgical work of the war had no doubts, and in the report of Surgeon-General Billings, in Circular 4 of the "Medical and Surgical History of the War," he says, speaking of hospital construction, "*The object to be kept in view is to furnish shelter without diminishing that supply of pure air and light which is necessary to health.*" Other points, such as locality, exposure, plan of construction, modes of heating and ventilation—are only of importance in so far as they secure that object. Wiser words were never written, but it is doubtful whether their meaning and importance have been fully appreciated or allowed due weight when the question of hospital construction has arisen. We have learnt that life in the open air is not only possible but pleasant—that cold air is a tonic, as George Bodington maintained—that warm rooms are debilitating, and that a free supply of external air is not disagreeable, while a current of air that enters from ventilators or partly opened windows and other narrow openings causes draughts which, when they impinge upon the inmates, give rise to discomfort and complaint too often resulting in their being stopped up.

### What Open Air Does in War-Time.

It is to be hoped that these principles may be borne in mind in the course of the arrangements that are being made and will have to be made in the provision for the sick and wounded during the present war. It is quite probable that the accommodation already made will prove insufficient. In the American Civil War and in the Franco-Prussian War the suitability of wooden buildings was abundantly proved, and with our increased knowledge and confidence in the advantages of simple structures it is to be hoped that no more money will be wasted upon structural alterations for hospital use, but that simple erections will be made, for which there are many good designs to be seen in the more recently erected sanatoria for the treatment of consumption, none being better than that recently opened at Little Bromwich, outside Birmingham. Even these in many respects may be simplified in accordance with the temporary use to which they will be put. Open wards dispense with all need for considering methods of heating and ventilation; windows and doors are not required, but it may be desirable in some places to have sliding wooden screens to afford a certain amount of protection in stormy weather. Flooring may be made of asphalt or simply beaten earth covered with a thick layer of pine sawdust, which can be renewed easily with a shovel and which absorbs all damp to an extraordinary extent. The late Professor George Vivian Poore demonstrated the great value of pine sawdust as an antiseptic absorbent and its special value for taking up and deodorising such liquids as urine. He was asked by the South-Western Railway to suggest a simple form of urinal for the use of the men engaged in their works at Basingstoke, and he proposed the provision of a simple trough made by two planks put at an angle, filled with pine sawdust; when he was asked how often the sawdust would have to be renewed he said "Practically never." He was in the habit of showing his friends a flannel jelly-bag half full of sawdust which he and his assistant had used in his room at University College for six months as a urinal, the effluent from which in the whole of that time amounted to something less than a pint of a pale fluid which smelt only of turpentine.

### The Proposed Temporary Hospitals.

If these hospitals are put up as is proposed in the grounds attached to buildings, or in unoccupied land in their neighbourhood, there ought to be no difficulty about the disposal of fæces, which should be superficially buried in shallow trenches. Soiled dressings should be burnt, and a proper furnace connected with the sterilising apparatus would be a necessary adjunct to the hospital. The University buildings at Bournebrook, Birmingham, have been transformed at very considerable expense into a hospital of 600 beds, but it is likely that many more will be needed. There is plenty of room for the erection of such an annexe as I have in mind, and there would be no difficulty in Birmingham in forming a committee to provide the money and equipment and to superintend the provision of all that is needed for such an extension. The campaign in favour of fresh air and sunlight is also that of efficiency and economy, and where money may be the decisive factor in this great struggle, there can be no justification for wasting it, especially when better results may be obtained by cheaper methods.

